# Preparation of *Artemisia argyi*-Derived Extracellular Nanovesicles and Their Protective Effects Against Oxidative Stress-Induced Senescence in Endometrial Stromal Cells

**DOI:** 10.3390/bioengineering13030256

**Published:** 2026-02-24

**Authors:** Xiudan Zheng, Rui Huang, Zhijun Liu, Tianfeng Liu, Han Lin, Lanlan Yin, Qiang Wu, Mingyan Zhao

**Affiliations:** 1 The State Key Laboratory of Mechanism and Quality of Chinese Medicine, Macau University of Science and Technology, Avenida Wai Long, Taipa, Macao SAR 999078, China; 3230005096@student.must.edu.mo (X.Z.);; 2 Department of Stem Cell Research and Cellular Therapy Center, Orthopedic Center, Affiliated Hospital of Guangdong Medical University, Zhanjiang 524001, China

**Keywords:** oxidative stress, plant-derived extracellular nanovesicles, ROS scavenging, cellular senescence

## Abstract

Oxidative stress-induced endometrial injury has been shown to contribute to infertility; however, effective strategies that can simultaneously scavenge reactive oxygen species (ROS) and restore mitochondrial and antioxidant homeostasis remain elusive. In this study, we isolated extracellular nanovesicles from *Artemisia argyi* (A-NVs) and investigated their protective effects on H_2_O_2_-damaged human endometrial stromal cells (hESCs). We discovered that A-NVs possess a typical lipid bilayer structure and contain a variety of bioactive components. Our metabolomic analysis indicates that A-NVs can be regarded as a “natural drug reservoir”, in which flavonoids account for approximately 10.8%. We demonstrate that A-NVs can be efficiently taken up by cells, improve cell viability, reduce intracellular and mitochondrial ROS levels, enhance superoxide dismutase (SOD) activity, upregulate the expression of catalase (*CAT*), *SOD1*, and *SOD2*, and partially restore mitochondrial membrane potential. Mechanistically, A-NVs exert antioxidant effects by activating the SIRT1/PGC-1α/Nrf2 signaling axis. SIRT1 activation further alleviates H_2_O_2_-induced premature senescence, as evidenced by a 71.8% reduction in SA-β-Gal-positive cells compared with the H_2_O_2_ group, together with downregulation of p53 and p21 expression. These positive protective effects can be blocked by the SIRT1 inhibitor EX-527, confirming the central role of this pathway. Collectively, our findings demonstrate that A-NVs can maintain redox and mitochondrial homeostasis while inhibiting oxidative stress-related senescence progression, underscoring their application potential in endometrial repair and functional recovery.

## 1. Introduction

Normal endometrial function relies on stromal cells, which undergo tightly regulated proliferation, differentiation, and decidualization across the menstrual cycle [1]. This dynamic remodeling imposes stringent demands on metabolic flexibility and the maintenance of cellular redox homeostasis [2]. Clinically, impaired endometrial receptivity and defective decidualization are widely recognized as important endometrial-factor contributors to female infertility, including unexplained infertility and repeated failure in assisted reproductive technologies (ART) [3]. Oxidative stress, arising from an imbalance between intracellular reactive oxygen species (ROS) production and antioxidant clearance, is a pivotal disruptive factor that compromises endometrial receptivity and contributes to implantation failure and recurrent pregnancy loss [4]. Persistent or excessive oxidative stress can further establish and sustain cellular senescence, typically accompanied by activation of the p53/p21 or p16^INK4a^–Rb axes and increased senescence-associated β-galactosidase (SA-β-gal) activity [5]. Meanwhile, oxidative stress-induced mitochondrial injury and impaired quality control such as insufficient mitochondrial biogenesis and defective mitophagy/mitochondrial clearance can drive sustained ROS generation, forming a feed-forward “ROS–mitochondrial damage–ROS” loop that shifts senescence from a transient stress response to a long-term steady-state alteration [6]. Under certain conditions, this process may further promote apoptotic programs, thereby disrupting endometrial tissue homeostasis [7]. Notably, senescent stromal cells can exhibit reduced decidual responsiveness and an altered secretory phenotype, which may perturb the endometrial microenvironment and thereby increase the risk of infertility-related outcomes such as recurrent implantation failure and pregnancy loss [8]. Although the role of oxidative stress in the initiation and progression of female reproductive disorders has attracted substantial attention, precise intervention strategies targeting upstream regulatory nodes remain to be fully explored.

In the search for safe and effective regulators from natural products, *Artemisia argyi* has attracted increasing attention owing to its traditional anti-inflammatory and antioxidant applications [9]. Previous studies have shown that *Artemisia argyi* extracts can reduce ROS levels in specific cellular models and modulate the expression of genes associated with antioxidant defense, stress responses, and fibrosis, thereby mitigating tissue injury [10,11]. In in vitro senescence-related cell models, an aqueous extract of *Artemisia argyi* has been reported to dose-dependently decrease mitochondrial superoxide levels, alleviate DNA damage and telomere-shortening-associated alterations, and show a trend toward reversing senescence-associated phenotypes such as p21 upregulation [12]. In addition, in an ovariectomized rat model, *Artemisia argyi* polysaccharides increased plasma estradiol levels, elevated relative uterine weight, and upregulated uterine estrogen receptor-α expression, suggesting a potential modulatory effect on reproductive tissues and endocrine function under estrogen-deficient conditions [13]. Consistent bioactivity and tissue-specific delivery are crucial for achieving reproducible improvements in endometrial function and receptivity. However, crude extracts and isolated phytochemicals often face practical limitations for translation, including compositional variability, limited stability, and insufficient efficiency of targeted delivery [14,15], thereby substantially constraining the effective utilization of *Artemisia argyi* bioactivity.

In recent years, plant-derived extracellular nanovesicles (PDNVs) have shown great promise in the fields of cross-species information transfer and therapeutic delivery. They are a new type of natural cell-free nanocarriers [16,17]. These lipid bilayer vesicles from plants usually carry a variety of biologically active components. These components include lipids, flavonoids and other phytochemicals, small RNAs/miRNAs, proteins and metabolites [18]. So, PDNVs have both carrier functions and inherent biological activities. Compared with synthetic nanoparticles, free phytochemicals or crude extracts, their vesicle structure has many advantages. It can enrich biologically active components, enhance physical and chemical stability, protect degradable molecules, and improve cell uptake efficiency and tissue targeting [19]. Importantly, accumulating studies have demonstrated that PDNVs can be produced and standardized with favorable biocompatibility and can be engineered or scaled for therapeutic delivery in vivo, supporting their translational potential as a cell-free nanomedicine platform [20,21]. In studies related to *Artemisia argyi*, oral administration of nanovesicles derived from *Artemisia argyi* (A-NVs) has been reported to relieve ulcerative colitis in mice [22], demonstrating the feasibility of isolating bioactive vesicles from this herb and achieving in vivo efficacy. Nevertheless, whether A-NVs can directly protect endometrial stromal cells (ESCs) from oxidative stress-driven senescence, and through which regulatory axis they exert these effects, remains to be elucidated.

Sirtuin 1 (SIRT1), an NAD^+^-dependent class III deacetylase, is regarded as a key molecular hub integrating metabolic sensing, oxidative stress responses, and tissue functional maintenance [23]. Mechanistically, SIRT1 promotes mitochondrial biogenesis and improves metabolic efficiency by deacetylating transcriptional coactivators such as PGC-1α [24]. In parallel, SIRT1 can reinforce Nrf2-driven antioxidant transcriptional programs and upregulate critical antioxidant enzymes, including superoxide dismutase 2 (SOD2), thereby enhancing ROS-scavenging capacity [25]. Beyond redox regulation, SIRT1 exerts multi-target control over the “senescence switch”: it can attenuate p53-driven cell-cycle arrest through deacetylation and modulate stress resilience via FOXO- and NF-κB-associated pathways, placing it at the nexus of the “oxidative injury–senescence–microenvironmental inflammation/fibrosis” axis [26,27]. In ESC biology, upregulation of SOD2 and Nrf2 during decidualization accompanies reduced intracellular ROS, whereas SIRT1 inhibition or knockdown exacerbates ROS accumulation—potentially in concert with inadequate antioxidant gene expression—underscoring the importance of SIRT1 in maintaining redox balance and stromal cell function [28,29]. Accordingly, restoring SIRT1-centered redox and mitochondrial homeostasis in ESC may represent a mechanistically grounded therapeutic strategy to improve endometrial receptivity, thereby benefiting the management of infertility, including outcomes in ART [28].

Based on the above rationale, this study isolated and characterized A-NVs and investigated their biological activity in an H_2_O_2_-induced oxidative injury model of human endometrial stromal cells (hESCs). We first evaluated vesicle properties and quality through physicochemical and morphological analyses, and then assessed cytoprotective effects by measuring cell viability, intracellular and mitochondrial ROS levels, mitochondrial functional readouts, and senescence-associated markers. In addition, to elucidate the mechanistic basis underlying the anti-senescence effects of A-NVs, we focused on the SIRT1/PGC-1α/Nrf2 signaling network, which integrates mitochondrial biogenesis, antioxidant transcriptional programs, and senescence regulation, and employed pharmacological blockade to verify the critical contribution of SIRT1 to A-NVs–mediated protection. Results are reported herein.

## 2. Materials and Methods

### 2.1. Extraction and Characterization of A-NVs

#### 2.1.1. Isolation of A-NVs

Fresh *Artemisia argyi* leaves were cut into small fragments (0.5–10 mm^2^) and homogenized in PBS buffer at a ratio of 1:1 g/mL. After incubation at 4 °C for 8–16 h, the mixture was filtered through medical gauze to remove plant debris and large impurities. The filtrate was collected and subjected to screening for the removal of residual particulates, and then centrifuged sequentially at 400× *g*, 4000× *g*, and 10,000× *g* for 30 min each to further clarify the suspension. The resulting supernatant was filtered through a 0.45 μm filter, followed by ultracentrifugation at 110,000× *g* for 60 min. The final pellet was resuspended in PBS and filtered through a 0.22 μm filter to obtain purified A-NVs. Protein concentration was quantified using a BCA assay kit (Beyotime, Shanghai, China). The morphology and structural characteristics of A-NVs were examined by transmission electron microscopy (TEM, JEM-1400, JEOL, Tokyo, Japan). Particle size distribution and zeta potential were determined using nanoparticle tracking analysis (Nanosight NS300, Malvern, UK) and a zeta potential analyzer (Nanocoulter G, Resuntech, Shenzhen, China), respectively. Lyophilized A-NVs and commercial *Artemisia argyi* extract were analyzed by FT-IR to detect and compare their infrared absorption spectra.

#### 2.1.2. Coomassie Brilliant Blue Staining

A-NVs were added into radioimmunoprecipitation (RIPA) lysis buffer (Thermo Fisher Scientific, Waltham, MA, USA) for ultrasonic lysis. Proteins from the lysate samples were separated by SDS-PAGE, followed by Coomassie brilliant blue staining visualization using a commercial Kit (Beyotime) according to the manufacturer’s instructions, and then photographed.

#### 2.1.3. Metabolomic Analysis

A 100 μL aliquot of A-NVs was transferred to a 2 mL centrifuge tube, followed by the addition of 200 μL of pre-chilled methanol:acetonitrile (1:1, *v*/*v*). After vortexing, freeze–thaw cycling, and centrifugation (12,000× *g*, 10 min, 4 °C), 200 μL of the supernatant was collected and dried under vacuum. The dried residue was reconstituted in 150 μL of 50% methanol containing 5 ppm 2-chlorophenylalanine (internal standard). After vortexing (30 s) and centrifugation (12,000 rpm, 10 min, 4 °C), the supernatant was filtered through a 0.22 μm membrane and subject to LC-MS analysis. Potential targets identified from metabolomic data were subjected to KEGG pathway analysis. Results were sorted by ascending log *p* values and visualized in a bubble plot.

### 2.2. Cell Culture

Human endometrial stromal cells (hESCs, P5-P10, Procell Life Science&Technology Co., Ltd., Wuhan, China) were cultured in DMEM/F12 complete medium (Gibco, Frederick, MD, USA) supplemented with 10% FBS and 1% penicillin-streptomycin at 37 °C under 5% CO_2_.

#### 2.2.1. Cellular Uptake Assay

A-NVs were fluorescently labeled with PKH26 (Sigma-Aldrich, St. Louis, MO, USA) according to the manufacturer’s instructions. To minimize potential artifacts arising from free-dye micelles/aggregates, unbound PKH26 was removed by repeated post-labeling purification/washing steps, and PKH26-labeled A-NVs were finally collected by ultracentrifugation at 110,000× *g* for 60 min [30]. hESCs were seeded at a density of 1 × 10^4^ cells per well onto sterile coverslips in 24-well plates. Cells were then incubated with PKH26-labeled A-NVs in Low-serum medium (1% FBS) for 24 h in the dark at 37 °C. After fixation with 4% paraformaldehyde, nuclei were stained with DAPI (Invitrogen, Waltham, MA, USA) for 5–10 min. Coverslips were mounted, and samples were observed under a confocal laser scanning microscope (CLSM, Zeiss LSM900, Jena, Germany).

#### 2.2.2. Effect of A-NVs on Proliferation of hESCs

hESCs were seeded into 96-well plates at 3 × 10^4^ cells/mL and allowed to attach overnight. Cells were then treated with A-NVs at 0, 5, 10, 25, 50, or 100 μg/mL in complete DMEM/F12 and cultured for 24, 48, and 72 h. Cell metabolic activity was measured using a CCK-8 kit (MedChemExpress, Monmouth Junction, NJ, USA) according to the manufacturer’s instruction.

#### 2.2.3. Protective Effect of A-NVs on Proliferation of Oxidatively Injured hESCs

To establish an in vitro oxidative injury model, hESCs were cultured for 24 h to allow complete attachment. Oxidative injury was then induced with H_2_O_2_, and cells were assigned to three groups with triplicate wells per condition: (1) Blank control group: Cells were incubated with fresh complete medium without H_2_O_2_ for 2 h; (2) H_2_O_2_ model group: Cells were incubated with fresh complete medium containing 200 μM H_2_O_2_ [31] at 37 °C and 5% CO_2_ for 2 h; (3) A-NVs treatment group: After H_2_O_2_ exposure, the medium was discarded, and cells were gently washed twice with PBS, followed by incubation with complete medium containing A-NVs at concentrations of 0, 5, 10, 25, 50, or 100 μg/mL. After modeling and treatment, cell metabolic activity was assessed using the CCK-8 assay at 24, 48, and 72 h of culture.

#### 2.2.4. SOD Activity Assay

To investigate whether A-NVs can restore oxidative damage and enhance cellular SOD activity in hESCs, SOD activity was measured using a commercial detection kit (Abbkine, Wuhan, China). hESCs were seeded in 6-well plates at a density of 1 × 10^5^ cells per well. After overnight culture, oxidative stress was induced as described above. Following H_2_O_2_ exposure, the medium was replaced with fresh complete medium containing different concentrations of A-NVs (5, 25, 50, and 100 μg/mL). After incubation for an additional 48 h, the cells were collected, resuspended in 1× lysis buffer, and disrupted by ultrasonication. The resulting supernatant was harvested, and SOD activity was determined following the manufacturer’s instructions. The absorbance was measured at 450 nm using a microplate reader (BioTek, Winooski, VT, USA).

#### 2.2.5. Intracellular ROS Measurement

To evaluate ROS scavenging capacity of A-NVs, the intracellular ROS level was evaluated using the fluorescent probe 2′,7′-dichlorodihydrofluorescein diacetate (DCFH-DA, Beyotime). hESCs were seeded in 12-well plates at a density of 5 × 10^4^ cells per well. After overnight culture and H_2_O_2_ treatment, cells were incubated with A-NVs (25 and 50 μg/mL) in complete medium for 48 h. Thereafter, cells were stained with DCFH-DA for 20–25 min at room temperature in the dark, followed by nuclear staining with DAPI. Fluorescence images were acquired by a fluorescence microscope (OLYMPUS, Hachioji, Japan). Quantitative analysis of ROS fluorescence intensity was performed by ImageJ (version 1.53k).

To specifically assess mitochondrial ROS levels, cells were stained with the mitochondrial-targeted probe MitoSOX Red (MCE) under the same cell culture and oxidative stress conditions. Images were captured using a fluorescence microscope, and mitochondrial ROS (mtROS) levels were quantified with ImageJ.

#### 2.2.6. Evaluation of Mitochondrial Membrane Potential

Mitochondrial membrane potential (MMP) was detected using the JC-1 Membrane Potential Detection Kit (Solarbio, Beijing, China). hESCs were seeded in 12-well plates or onto coverslips at a density of 5 × 10^4^ cells per well, with or without H_2_O_2_ pretreatment. After treatment with A-NVs (25 and 50 μg/mL) in complete medium for 48 h, cells were stained with JC-1 according to the manufacturer’s instruction. Fluorescence images were acquired under a fluorescence microscope and analyzed using ImageJ.

#### 2.2.7. SA-β-Gal Staining

Cellular senescence was assessed using a SA-β-gal staining kit (Beyotime). Following the same oxidative stress modeling and A-NVs intervention treatment described above, hESCs were fixed with 4% paraformaldehyde for 15 min at room temperature and washed three times with PBS. Cells were then incubated with SA-β-gal staining solution at 37 °C overnight. The presence of blue cytoplasmic granules was considered a positive signal for senescence, and images were captured under an inverted microscope (OLYMPUS, Japan).

#### 2.2.8. Quantitative Reverse Transcription Polymerase Chain Reaction (qRT-PCR)

Total RNA was extracted from treated cells using TRIzol reagent (Invitrogen, USA) and reverse transcribed into cDNA using HiScript III RT SuperMix (+gDNA wiper, Vazyme, Nanjing, China). qRT-PCR was performed using ChamQ Universal SYBR qPCR Master Mix (Vazyme) on a real-time PCR system (Applied Biosystems QuantStudio Dx, Waltham, MA, USA). The mRNA expression levels of *CAT*, *SOD1*, and *SOD2* were normalized to β-actin as the internal reference gene using 2^−ΔΔCt^ method [32]. Primer sequences are shown in Table 1.

#### 2.2.9. SIRT1 Pathway Inhibition

To investigate the role of SIRT1 in the protective effects mediated by A-NVs, the SIRT1-specific inhibitor EX-527 (Selisistat; MCE, Monmouth Junction, NJ, USA) was used to block the SIRT1 signaling pathway. EX-527 was initially prepared as a stock solution in dimethyl sulfoxide (DMSO) and subsequently diluted in the culture medium to a final concentration of 10 μM. Cells were further divided into four groups: (1) blank control, (2) H_2_O_2_, (3) H_2_O_2_ + A-NVs, and (4) H_2_O_2_ + EX-527 + A-NVs. After H_2_O_2_-induced oxidative injury, cells were washed twice with PBS and incubated in fresh medium supplemented with EX-527 (10 μM) and A-NVs (50 μg/mL) at 37 °C and 5% CO_2_ for 48 h before further analyses.

#### 2.2.10. Western Blotting

hESCs were collected and lysed in RIPA lysis buffer. Equal amounts of proteins from lysis samples were separated via SDS-PAGE and then transferred onto PVDF membranes (Merck Milipore, Burlington, MA, USA). After rinsing with TBST (Solarbio), membranes were blocked with 5% skimmed milk (Becton, Franklin Lakes, NJ, USA) at room temperature for 1 h. The membranes were then incubated overnight at 4 °C with the following primary antibodies (all from Proteintech, Rosemont, IL, USA) at a dilution of 1:5000 anti-SIRT1, anti-PGC-1α, anti-Nrf2, anti-p53, anti-p21, and anti-β-actin. Following thorough washing with TBST, the membranes were incubated with horseradish peroxidase (HRP)-conjugated secondary antibody (1:5000, Proteintech) at room temperature for 1 h. Protein bands were visualized using chemiluminescence (ECL) reagent (Zeta Life, Menlo Park, CA, USA) and imaged with the BG-gdsAUTO720 Gel Imaging System (Baygene Biotech Company Limited, Beijing, China).

#### 2.2.11. Immunofluorescence Staining

hESCs were seeded onto sterilized coverslips in 24-well plates at a density of 2 × 10^4^ cells. After overnight culture and the indicated treatments, cells were fixed with 4% paraformaldehyde for 30 min, permeabilized with 0.1% Triton X-100 for 20 min, and blocked with 1% bovine serum albumin for 30 min at room temperature. Cells were then incubated overnight at 4 °C with primary antibodies against SIRT1 (1:200, Proteintech) and Nrf2 (1:200, Proteintech). After washing, samples were incubated with Alexa Fluor 488-conjugated secondary antibody (1:200) for 40 min. Coverslips were mounted using an anti-fade mounting medium containing DAPI. Images were acquired using a CLSM.

### 2.3. Statistical Analysis

All data are expressed as mean ± standard deviation from n = 3 independent biological experiments. Statistical significance was analyzed using GraphPad Prism 8. Comparisons between two groups were conducted using Student’s *t*-test, while multiple group comparisons were performed using one-way analysis of variance (ANOVA). All experiments were repeated at least three times. The levels of statistical significance are indicated as follows: * *p* < 0.05, ** *p* < 0.01, *** *p* < 0.001 and **** *p* < 0.0001.

## 3. Results

### 3.1. Preparation and Characterization of A-NVs

**Figure 1:**
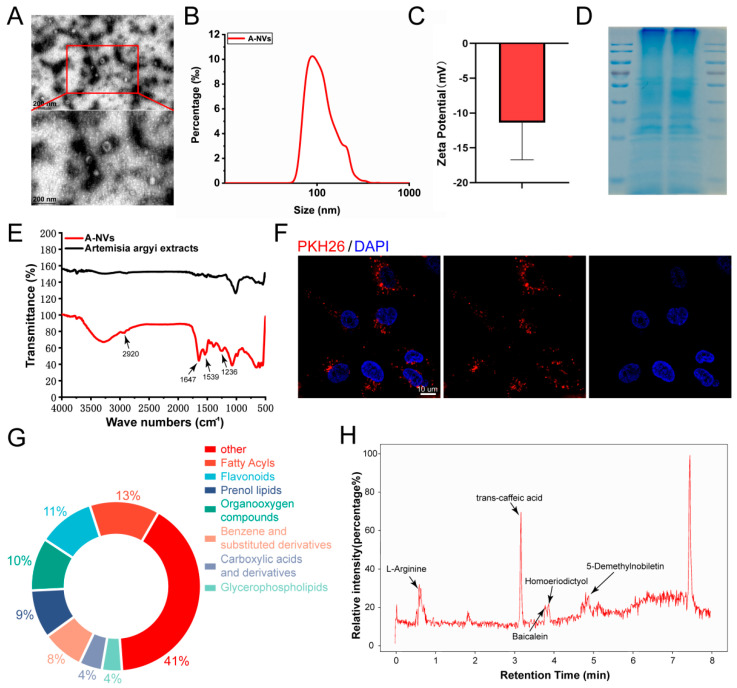
Characterization of *Artemisia argyi*-derived extracellular nanovesicles (A-NVs). (**A**) Representative TEM image of A-NVs. The particle size distribution (**B**) and zeta-potential measurements (**C**) of A-NVs. (**D**) Protein profile of A-NVs analyzed by SDS-PAGE and Coomassie brilliant blue staining. (**E**) FT-IR spectra of A-NVs and commercial *Artemisia argyi* extract. (**F**) Fluorescence images show the uptake of A-NVs by hESCs. Cells were incubated with PKH26-labeled A-NVs (red) for 24 h, nuclei were counterstained with DAPI (blue). (**G**) Composition analysis of metabolites the identified in A-NVs by untargeted metabolomics. (**H**) The LC-MS spectrum of A-NVs.

A-NVs were isolated through sequential centrifugation and filtration. As shown in Figure 1A, A-NVs exhibited a typical round or cup-shaped morphology with a discoid concave structure, and had an average size of approximately 120 nm. NTA revealed a size ranging from about 60.5 to 241.5 nm (Figure 1B). NTA indicated that the particle concentration in the final A-NV suspension was 2.4 × 10^10^ particles/mL. Based on 100 g of starting tissue, the final 5 mL suspension contained an estimated total of 1.2 × 10^11^ particles, corresponding to a yield of 1.2 × 10^9^ particles per gram of tissue. Protein quantification by the BCA assay showed a protein concentration of 8.7 mg/mL, and the particle-to-protein ratio was calculated to be 2.76 × 10^6^ particles/µg protein. Zeta potential measurements showed that the zeta potential of A-NVs was approximately −14.5 mV (Figure 1C), which lies within the typical surface charge range of nanovesicles [22,33]. SDS-PAGE revealed the presence of proteins with molecular weights between 10 and 250 kDa (Figure 1D). FT-IR analysis demonstrated that, compared to commercial *Artemisia argyi* extracts, A-NVs exhibited characteristic absorption peaks at 1539 cm^−1^ and 1647 cm^−1^, corresponding to C=O stretching vibrations (amide I) and combined N-H bending/C-N stretching vibrations (amide II), respectively, which are indicative of peptide bonds in proteins. The peak at 2920 cm^−1^ represented asymmetric CH_2_ stretching vibrations, characteristic of long-chain alkanes in lipid molecules, supporting the lipid bilayer membrane structure of extracellular vesicles. Additionally, a peak at 1236 cm^−1^ corresponded to asymmetric P=O stretching vibrations, reflecting phosphodiester bonds typically associated with nucleic acids (RNA/DNA) carried within nanovesicles (Figure 1E).

Metabolomics analysis revealed that A-NVs possessed diverse components, including 52 fatty acyls (13%), 42 flavonoids (10.8%), 39 organooxygen compounds (10%), as well as various prenol lipids and glycerophospholipids (Figure 1G). Notably, these components contained multiple flavonoids with well-documented anti-inflammatory and antioxidant properties, such as homoeriodictyol, baicalein, and 5-demethylnobiletin (Figure 1H).

### 3.2. Cellular Uptake and Cytocompatibility of A-NVs

Successful cellular internalization of nanovesicles is a prerequisite for their functional activity. Fluorescence imaging confirmed that PKH26-labeled A-NVs were effectively taken up by hESCs (Figure 1E).

**Figure 2:**
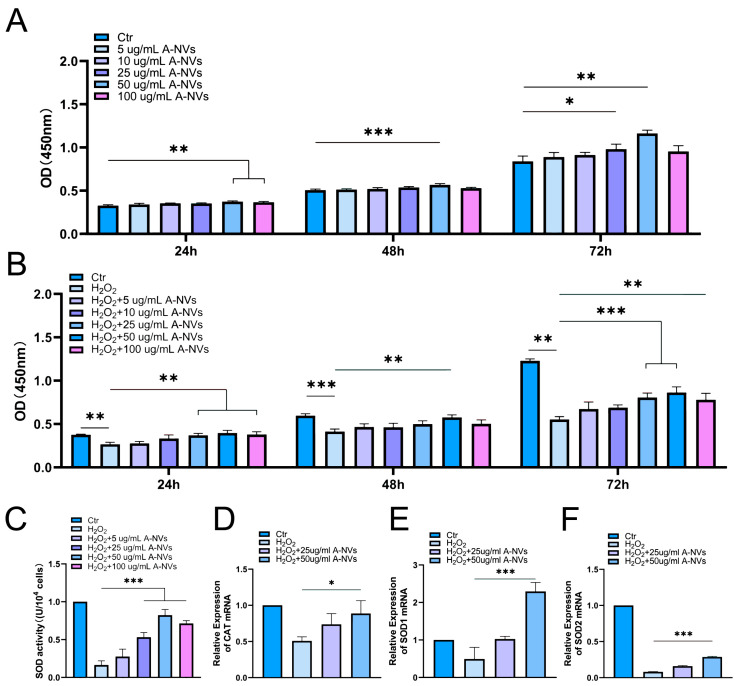
A-NVs protect hESCs from H_2_O_2_-induced oxidative damage. (**A**) Proliferation of hESCs treated with different concentrations of A-NVs for 24, 48 and 72 h, as determined by the CCK-8 assay. (**B**) Proliferation of hESCs exposed to H_2_O_2_ and then treated with A-NVs for 24, 48, and 72 h. (**C**) SOD Activity in hESC under the indicated treatments. (**D**–**F**) The mRNA expression of *CAT*, *SOD1*, and *SOD2* in hESCs measured by qRT-PCR. (Data were shown as mean ± SD; * *p* < 0.05, ** *p* < 0.01, *** *p* < 0.001, n = 3).

To investigate the biocompatibility of A-NVs in vitro, hESCs were incubated with different concentrations (5, 10, 25, 50, 100 μg/mL) of A-NVs, and cell proliferation was assessed using the CCK-8 assay kit. The results demonstrated that A-NVs did not exhibit any cytotoxic effects on hESCs (Figure 2A). Within the concentration range of 25–100 μg/mL, A-NVs promoted cell proliferation, with the most notable effect observed at 50 μg/mL. Subsequently, A-NVs at concentrations of 25 and 50 μg/mL were selected for subsequent experiments.

### 3.3. A-NVs Attenuate H_2_O_2_-Induced Oxidative Damage in hESCs

An oxidative damage model was established by treating hESCs with 200 μM H_2_O_2_ for 2 h. As shown in Figure 2B that cell metabolic activity was markedly reduced following H_2_O_2_ exposure. After A-NVs treatment, cell proliferative activity was enhanced with increasing A-NVs concentrations, showing a concentration-dependent protective effect, which alleviated H_2_O_2_-induced injury. Notably, the strongest protective effect was observed at 50 μg/mL after 72 h of incubation.

To further elucidate the mechanism underlying the antioxidative effect of A-NVs, we evaluated the activity and expression of key endogenous antioxidant enzymes in hESCs. As shown in Figure 2C, compared with the control group, H_2_O_2_ exposure significantly inhibited SOD (a key enzyme responsible for catalyzing the dismutation of superoxide anions) activity by approximately 82%, indicating severe impairment of the primary cellular antioxidant defense. Notably, A-NVs treatment restored SOD activity in a dose-dependent manner, with the activity recovered to near-normal levels at a concentration of 50 μg/mL.

We next investigated whether this restoration of enzyme activity was associated with changes in gene expression. qRT-PCR analysis revealed that H_2_O_2_-induced oxidative stress significantly downregulated the mRNA levels of several key antioxidant enzymes, including *CAT*, *SOD1*, and *SOD2* (Figure 2D–F). In contrast, A-NVs treatment significantly upregulated the expression levels of these genes compared with the H_2_O_2_-only group, consistent with the observed recovery of SOD activity.

### 3.4. A-NVs Scavenge H_2_O_2_-Induced Intracellular ROS in hESCs

**Figure 3:**
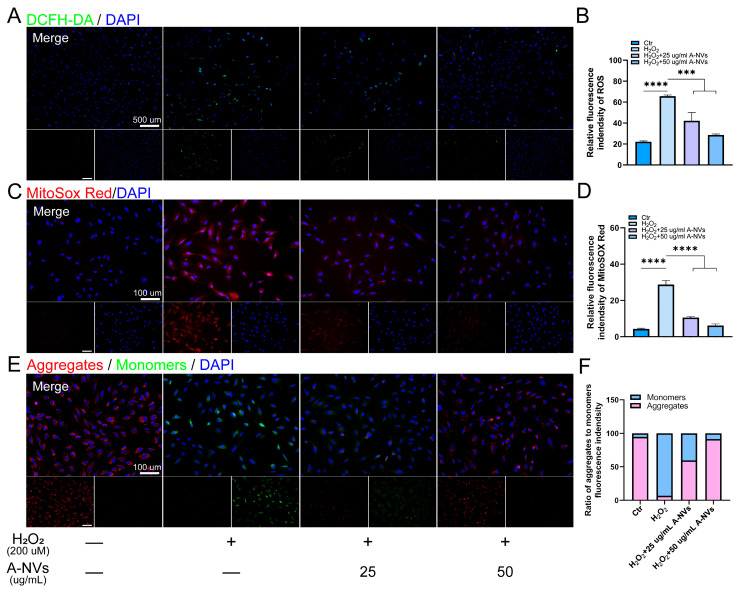
Antioxidant effects of A-NVs in hESCs under oxidative stress. (**A**) Intracellular ROS levels detected by DCFH-DA fluorescence in hESCs after H_2_O_2_ stimulation and A-NVs treatment. (**B**) Quantitative fluorescence intensity analysis of ROS. (**C**) mtROS staining in hESCs incubated with A-NVs under oxidative stress. (**D**) Quantitative analysis of mtROS fluorescence intensity. (**E**) Fluorescence images of MMP detected by JC-1 staining under different treatments. (**F**) Red/green fluorescence intensity ratio corresponding to JC-1 aggregate/monomer forms. (Data were shown as mean ± SD; *** *p* < 0.001, **** *p* < 0.0001, n = 3).

To directly visualize the antioxidant effects of A-NVs, we employed the fluorescent probe DCFH-DA to monitor intracellular ROS levels. As shown in Figure 3A,B, intense green fluorescence was observed in hESCs following H_2_O_2_ stimulation, indicating substantial ROS accumulation. Notably, treatment with A-NVs significantly attenuated H_2_O_2_-induced fluorescence signal in a concentration-dependent manner. Compared with the H_2_O_2_ group, both 25 μg/mL and 50 μg/mL A-NVs treatment reduced intracellular ROS levels in hESCs, with the 50 μg/mL A-NVs showing a more pronounced effect. Subsequently, we used the MitoSox Red-specific probe to detect mtROS levels in hESCs and found that the mtROS levels in the A-NV-treated groups were significantly lower than those in the H_2_O_2_ group (Figure 3C,D). Meanwhile, the antioxidant activity of A-NVs enabled direct scavenging of existing mtROS, creating favorable conditions for MMP recovery. JC-1 probe analysis revealed that A-NVs restored the aggregate/monomer ratio to 59% of the normal group (Figure 3E,F), indicating partial recovery of MMP.

### 3.5. A-NVs Activate the SIRT1/PGC-1α/Nrf2 Pathway in H_2_O_2_-Induced hESCs

**Figure 4:**
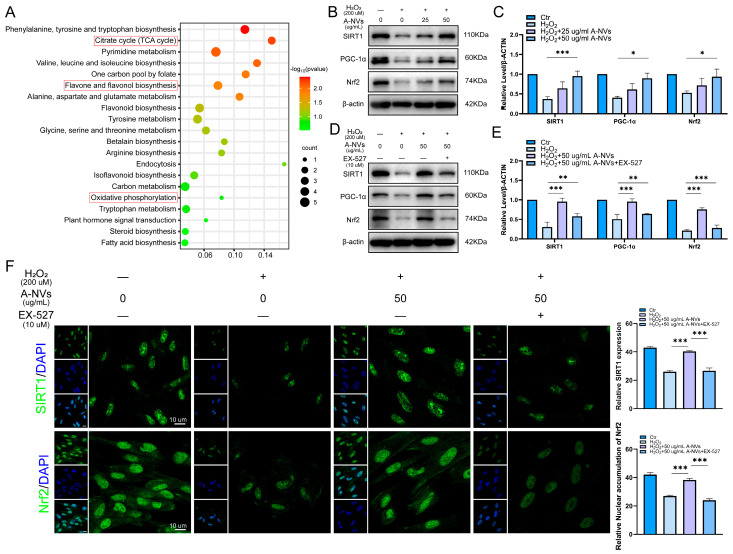
A-NVs protect hESCs from oxidative stress via the SIRT1/PGC-1α/Nrf2 pathway. (**A**) KEGG pathway enrichment analysis based on the metabolomic profile of the A-NVs. (**B**,**C**) Western blot analysis of SIRT1, PGC-1α, and Nrf2 protein levels in hESCs exposed to H_2_O_2_ (200 μM) and subsequently treated with A-NVs. (**D**,**E**) Western blot analysis of the protein levels of SIRT1, PGC-1α, and Nrf2 in hESCs after exposure to H_2_O_2_ followed by treatment with A-NVs and the SIRT1 inhibitor EX-527. (**F**) Immunofluorescence staining of SIRT1 and Nrf2 in hESCs across different treatment groups. (Data were shown as mean ± SD; * *p* < 0.05, ** *p* < 0.01, *** *p* < 0.001, n = 3).

To elucidate the molecular mechanisms underlying A-NVs-mediated enhancement of antioxidant defense in hESCs, we performed KEGG enrichment analysis on metabolomic data. The results showed that A-NVs were enriched in metabolites associated with oxidative stress-related pathways, such as oxidative phosphorylation, the citrate cycle and flavonol biosynthesis (Figure 4A). Subsequently, we evaluated the SIRT1/PGC-1α/Nrf2 signaling pathway using Western blot analysis. As shown in Figure 4B,C, H_2_O_2_ stimulation significantly downregulated the protein expression levels of SIRT1 and its key downstream effector PGC-1α, accompanied by a decrease in total Nrf2 expression. In contrast, A-NV treatment dose-dependently reversed the H_2_O_2_-induced downregulation of these proteins. Specifically, with the 50 μg/mL treatment restored the expression levels of SIRT1, PGC-1α, and total Nrf2 to near-normal levels.

### 3.6. SIRT1 Inhibitor Diminishes A-NVs-Mediated Protection of hESCs Against H_2_O_2_ Exposure

To further confirm the role of SIRT1 in A-NVs-mediated protective mechanisms, cells were treated with the specific SIRT1 inhibitor EX-527. As shown in Figure 4D,E, EX-527 effectively blocked the A-NVs-mediated upregulation of SIRT1 protein. Consequently, the A-NVs-mediated increases in PGC-1α and Nrf2 expression were markedly attenuated. Immunofluorescence analysis further supported these findings (Figure 4F): the enhanced nuclear accumulation of Nrf2 and the elevated SIRT1 fluorescence signal triggered by A-NVs were both diminished upon EX-527 co-treatment. These results indicate that pharmacological inhibition of SIRT1 reverses the antioxidant and pro-survival benefits conferred by A-NVs, underscoring that activation of the SIRT1/PGC-1α axis is indispensable for the protective effects of A-NVs against oxidative stress in hESCs.

### 3.7. A-NVs Alleviate H_2_O_2_-Induced Senescence in hESCs

**Figure 5:**
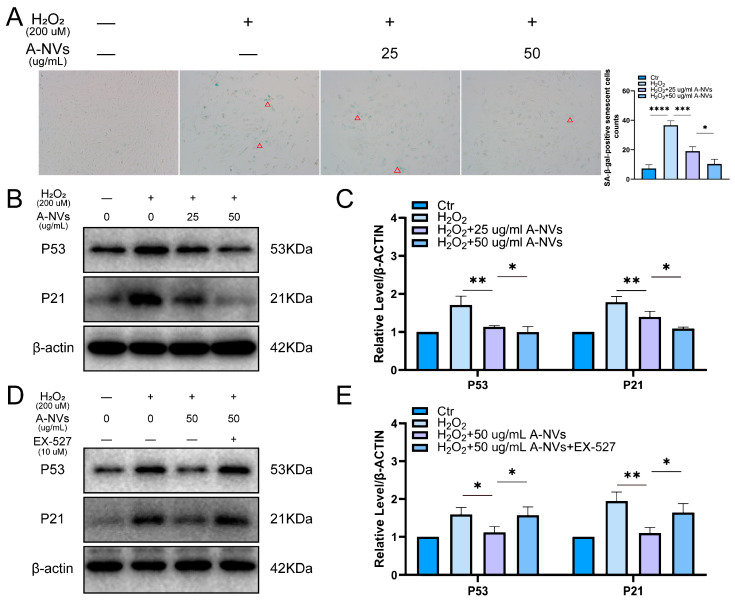
A-NVs attenuated hESCs senescence induced by H_2_O_2_. (**A**) Representative images of SA-β-gal staining in hESCs under indicated treatments and quantification of SA-β-gal-positive senescent cells count. Red triangles indicate SA-β-gal-positive senescent cells. (**B**,**C**) Western blot analysis of p53 and p21 protein levels in hESCs exposed to H_2_O_2_ and subsequently treated with A-NVs. (**D**,**E**) Western blot analysis of p53 and p21 in hESCs after exposure to H_2_O_2_ followed by treatment with A-NVs and the SIRT1 inhibitor EX-527. (Data were shown as mean ± SD; * *p* < 0.05, ** *p* < 0.01, *** *p* < 0.001, **** *p* < 0.0001, n = 3).

To investigate whether A-NVs can alleviate oxidative stress-associated senescence, SA-β-Gal staining was performed. As shown in Figure 5A, compared with the control group, H_2_O_2_ exposure resulted in a marked increase in the number of SA-β-Gal-positive cells. Notably, A-NV treatment significantly reduced the number of SA-β-Gal-positive cells, by 71.8% relative to the H_2_O_2_ group. Consistently, the expression levels of senescence-associated proteins p53 and p21 were decreased in hESCs after A-NV treatment compared with the H_2_O_2_ group (Figure 5B,C), indicating that A-NVs effectively alleviated H_2_O_2_-induced cellular senescence in hESCs. To further confirm the role of SIRT1 in this process, we detected the expression of p53 and p21 in hESCs treated with a SIRT1 inhibitor (EX-527). As shown in Figure 5D,E, EX-527 effectively abrogated the inhibitory effect of A-NVs on p53 and p21 expression (Figure 5D,E). These results demonstrate that A-NVs activate SIRT1 signaling pathway to attenuate the senescence process of hESCs.

## 4. Discussion

Oxidative stress is a central pathogenic factor in a variety of diseases and tissue injuries, and accumulating evidence indicates that it plays a pivotal role in endometrial injury and uterine dysfunction [34]. Consequently, intervention strategies aimed at enhancing endogenous antioxidant defense capabilities possess significant therapeutic value [35]. In this context, traditional herbal plants and their derived extracellular nanovesicles have emerged as a research hotspot in the field of nanotherapeutics in recent years [36], owing to their favorable biocompatibility, low immunogenicity, and intrinsic anti-inflammatory and antioxidant potentials. As a traditional medicinal plant, *Artemisia argyi* has attracted widespread attention for its biological activities in anti-inflammation, antioxidation, and tissue repair. Focusing on the cytoprotective effects of *Artemisia argyi*, this study systematically investigates the ability of its nanovesicles (A-NVs) to restore the endogenous antioxidant defense system, delay cellular senescence, and maintain cell viability. By targeting hESC oxidative stress and senescence, A-NVs may help improve the implantation-supportive endometrial microenvironment, thereby offering potential translational relevance for infertility treatment [37]. These findings provide a novel experimental basis for therapeutic strategies utilizing natural nanovesicles to repair oxidative endometrial injury.

Our physicochemical and morphological analyses confirmed the successful isolation of A-NVs exhibiting canonical nanovesicle features, including a lipid bilayer architecture and enrichment of protein and nucleic acid constituents—providing the structural and molecular basis for their bioactivity. Importantly, metabolomic profiling further supports A-NVs as a “natural pharmaceutical reservoir”: flavonoids comprise a substantial proportion of the functional cargo pool (~10.8%) and, together with lipids, amino acids, and terpenoids, form a multi-component system with potential synergistic actions. Among these, several identified flavonoids (e.g., homoeriodictyol, baicalein, and 5-demethylnobiletin) have been widely reported to possess antioxidant and anti-inflammatory properties [38,39]. As lipid-bilayer vesicles, A-NVs can effectively encapsulate and protect these constituents, thereby improving physicochemical stability and facilitating intracellular delivery compared with free compounds or crude extracts [18]. This vesicle-centered formulation thus provides a rational bridge for translating “plant bioactivity” into a more controllable, bioavailable therapeutic modality.

After establishing vesicle identity and compositional features, we next validated biological activity at the cellular level. A-NVs were efficiently internalized by hESCs and exhibited no cytotoxicity within the tested concentration range, supporting their cellular compatibility. In an H_2_O_2_-induced oxidative stress model, A-NVs exerted marked cytoprotective effects and restored cell viability in a concentration-dependent manner. DCFH-DA staining showed that this functional rescue was accompanied by a pronounced reduction in excessive intracellular ROS, consistent with re-establishment of redox homeostasis. Because ROS directly regulate redox-sensitive signaling, metabolism, and cell-fate programs, restoration of intracellular redox balance is tightly linked to recovery of cellular survival and proliferative capacity under stress [40].

Mechanistically, A-NVs reinforced endogenous antioxidant defenses at multiple levels. They enhanced superoxide detoxification by increasing SOD activity and simultaneously induced transcriptional upregulation of *CAT* and *SOD1/2*, indicating coordinated strengthening of the enzymatic antioxidant network. The concerted induction of these tightly transcriptionally regulated enzymes suggests that A-NVs do not act solely through direct chemical scavenging, but rather may activate upstream regulatory programs that orchestrate a sustained antioxidant response, ultimately achieving a more systemic reduction in intracellular ROS.

Given that mitochondrial dysfunction is both a consequence and an amplifier of oxidative stress, we further examined mitochondrial redox status and function. MitoSOX Red assays demonstrated that A-NVs significantly reduced mitochondrial ROS, which likely created favorable conditions for recovery of MMP. Consistently, JC-1 analysis revealed partial restoration of the aggregate/monomer ratio to approximately 59% of that in the normal group, indicating substantive recovery of MMP. Collectively, these findings support a model in which A-NVs reduce both global and mitochondrial oxidative burden and promote partial restoration of mitochondrial function, thereby potentially interrupting the feed-forward “ROS–mitochondrial damage–ROS” loop, helping preserve energy homeostasis and limiting oxidative stress-driven functional deterioration. Mitochondrial homeostasis and metabolic adaptation are closely linked to endometrial function, including stromal decidualization and endometrial receptivity [1,7], limiting oxidative stress-associated mitochondrial ROS accumulation may help preserve implantation competence in infertility-related settings characterized by oxidative and metabolic disturbances [2,7].

KEGG enrichment analysis implicated pathways such as oxidative phosphorylation and flavonoid biosynthesis, directing mechanistic investigation toward mitochondrial regulation and antioxidant programming. Given that the SIRT1–PGC-1α axis is a central hub of metabolic and redox homeostasis and that PGC-1α can cooperate with Nrf2 to amplify antioxidant transcriptional programs, we therefore focused on the SIRT1/PGC-1α/Nrf2 pathway.

Western blotting and immunofluorescence consistently showed that A-NVs reversed H_2_O_2_-induced suppression of SIRT1 and PGC-1α and promoted Nrf2 nuclear translocation. In this framework, SIRT1 serves as a sensor of cellular energy status and oxidative stress, and its activation enhances PGC-1α activity. PGC-1α, as a transcriptional co-activator supporting mitochondrial biogenesis and antioxidant gene expression, can act synergistically with Nrf2 to amplify antioxidant responses [41]. These results indicate that A-NVs initiate a SIRT1-centered regulatory program that couples mitochondrial regulation with antioxidant transcription, thereby conferring cytoprotection.

To determine whether SIRT1 is required for the observed effects rather than merely associated with them, we performed pharmacological loss-of-function experiments using the selective SIRT1 inhibitor EX-527. SIRT1 blockade markedly attenuated A-NVs-induced activation of the PGC-1α/Nrf2 axis and prevented Nrf2 nuclear translocation, indicating that the antioxidant response elicited by A-NVs is SIRT1 dependent.

Beyond acute cytoprotection, our data indicate that A-NVs also delay stress-induced cellular senescence—a consequence of sustained oxidative injury characterized by ROS accumulation, mitochondrial dysfunction, and DNA damage responses that ultimately converge on cell-cycle arrest programs such as p53–p21 [42]. A-NVs markedly reduced the number of SA-β-Gal-positive cells and downregulated senescence-associated proteins p53 and p21 in oxidatively injured human endometrial stromal cells, indicating attenuation of H_2_O_2_-induced senescence. Crucially, EX-527 completely abolished the inhibitory effects of A-NVs on p53 and p21, demonstrating that A-NVs retard senescence progression via a shared, SIRT1-dependent mechanism. Together, these findings link vesicle-mediated restoration of redox–mitochondrial homeostasis to functional suppression of senescence programming. Importantly, hESC senescence and p53/p21-driven cell-cycle arrest have been linked to impaired decidualization and a perturbed endometrial microenvironment, which can compromise implantation and contribute to infertility phenotypes such as recurrent implantation failure and pregnancy loss [43]. In this context, our finding that A-NVs alleviate oxidative stress-induced senescence through a SIRT1-dependent mechanism suggests a potential route to improve endometrial receptivity.

Nevertheless, several limitations should be acknowledged. Although we have identified flavonoids as major components, whether a single constituent plays a dominant role or multiple components act synergistically requires further studies. Moreover, the in vivo distribution, metabolic fate, and therapeutic efficacy of A-NVs in animal models of endometrial injury remain important topics for future research.

## 5. Conclusions

In summary, we successfully isolated *Artemisia argyi*-derived extracellular nanovesicles (A-NVs) and using an in vitro H_2_O_2_-induced oxidative damage model, demonstrated that A-NVs mitigate oxidative stress, thereby improving cell viability and delaying cellular senescence in hESCs. This positive protection is mediated by activation of the SIRT1/PGC-1α/Nrf2 signaling axis and enhancement of endogenous antioxidant defenses. Our findings highlight plant-derived nanovesicles as a natural, efficient, and multitargeted nanotherapeutic strategy, thus providing a novel theoretical and experimental foundation for the development of interventions targeting oxidative stress-related gynecological disorders, such as endometriosis, thin endometrium, and reproductive aging.

## Figures and Tables

**Table 1:** Primer sequences for qRT-PCR analysis of the relevant genes.

Gene	Forward	Reverse
*CAT*	GTGCGGAGATTCAACACTGCCA	CGGCAATGTTCTCACACACAGACG
*SOD1*	CTCACTCTCAGGAGACCATTGC	CCACAAGCCAAACGACTTCCAG
*SOD2*	CTGGACAAACCTCAGCCCTAAC	AACCTGAGCCTTGGACACCAAC
*β-actin*	CTCCATCCTGGCCTCGCTGT	GCTGTCACCTTCACCGTTCC

## Data Availability

The original contributions presented in the study are included in the article, further inquiries can be directed to the corresponding author.
